# Pathogenic Effector Th2 Cells in Allergic Eosinophilic Inflammatory Disease

**DOI:** 10.3389/fmed.2017.00165

**Published:** 2017-10-06

**Authors:** Alyssa Mitson-Salazar, Calman Prussin

**Affiliations:** ^1^Department of Immunobiology, Yale University School of Medicine, New Haven, CT, United States; ^2^Knopp Biosciences LLC, Pittsburgh, PA, United States

**Keywords:** Th2, CD161, CD294, chemoattractant receptor-homologous molecule expressed on Th2 cells positive, hematopoietic prostaglandin D synthase, interleukin-5, eosinophilic inflammation, eosinophilic gastrointestinal disease

## Abstract

There is an absolute requirement for Th2 cells in the pathogenesis of allergen-driven eosinophil-rich type 2 inflammation. Although Th2 cells are generally regarded as a homogeneous population, in the past decade there has been increasing evidence for a minority subpopulation of IL-5+ Th2 cells that have enhanced effector function. This IL-5+ Th2 subpopulation has been termed pathogenic effector Th2 (peTh2), as it exhibits greater effector function and disease association than conventional Th2 cells. peTh2 cells have a different expression profile, differentially express transcription factors, and preferentially use specific signaling pathways. As such, peTh2 cells are a potential target in the treatment of allergic eosinophilic inflammation. This review examines peTh2 cells, both in mouse models and human disease, with an emphasis on their role in the pathogenesis of allergic eosinophilic inflammation.

## Introduction and Early Observations

Since the first observation of cytokine heterogeneity of effector T helper (Th) cells (1), there have been attempts to examine the veracity of the Th1/Th2 paradigm and apply it to disease pathogenesis and treatment (1). Although initial investigations into this dichotomy in humans suggested a clearly laid out Th1–Th2 polarity (1), subsequent investigations were less clearly dichotomous (2). T cell biology is clonal, and as such, there are clear advantages to studying Th cell differentiation and cytokine expression at the single-cell level. The initial studies by Mossmann et al. (1) and the subsequent human investigations by Romagnani (3, 4) employed T cell clones, and although revolutionary at the time, had several technical limitations. T cell cloning is very labor intensive, limiting the number of clones (individual T cells) and patients who could be studied. More importantly, it is not clear that the cytokine phenotype of the resultant clone is the same as the original single T cell from which it was derived.

New scientific discovery is highly influenced by the development of new technology. During the mid-1990s intracellular cytokine staining was developed as a technique to interrogate the Th1/Th2 paradigm with greater fidelity and verisimilitude than possible with T cell cloning. Intracellular cytokine staining allows the examination of single-cell cytokine expression in thousands of individual cells, almost directly *ex vivo*. Initial publications clearly showed that although there was greater complexity in the Th1/Th2 paradigm than initially appreciated, the general paradigm was supported (5–7). Notably, in one of the authors’ (CP) early papers, IL-4 and IL-5 expression patterns differed, indicating that IL-5-producing cells were a minority subpopulation within the larger IL-4+ Th2 pool with a unique phenotype (CD27−, no IFN-γ coexpression) (5), which in a later review was hypothesized to be an IL-5+ Th2 subpopulation (8).

IL-5+ Th2 cell biology remained largely unexplored for the next 10 years, being relatively intractable to the available technology. The advent of polychromatic flow cytometry, and the ability to examine many phenotypic markers and cytokines within a single cell, facilitated subsequent murine and human investigations into IL-5+, pathogenic effector Th2 (peTh2) cell biology.

## Definitions and Identification of peTh2 Cells

Like many recently characterized cell populations, nomenclature has lagged behind the investigations of IL-5+ Th2 cells. Although the term “IL-5+ Th2” cells is probably the most commonly used descriptor, multiple other terms have been used. Clearly, a central distinguishing feature of these cells is their IL-5 expression. In our work in humans, we initially identified these cells by intracellular cytokine staining as IL-5+, IL-4+, IL-13+ relative to the IL-5− Th2 subpopulation that was IL-5−, IL-4+, IL-13+ (9). Subsequently, we demonstrated that the phenotypic markers hematopoietic prostaglandin D synthase (hPGDS) and CD161 individually identify IL-5+ Th2 cells (10). hPGDS in particular appears to be a more specific marker for pro-eosinophilic activity than IL-5 itself. To unify the various phenotypic descriptions, we have used the term peTh2, to emphasize the pathological role and enhanced effector function of these cells, which is due to more than simply IL-5 expression.

In parallel, Nakayama and colleagues have identified a murine peTh2 analog that they have termed “pathogenic memory” Th2, reviewed in Ref. (11). Their work has used an adoptive transfer memory model to generate IL-5+ Th2 and hence their terminology underscores the memory aspects of the model system. Luster and colleagues have identified a similar IL-5+ Th2 subpopulation during investigations to identify CCL8-responding T cells (12, 13). Lastly, Wambre and colleagues using class II tetramers have identified a subpopulation of human allergen-specific Th2 cells, which they have termed “Th2A” cells. Using both flow cytometric and transcriptome profiling, they characterize Th2A cells having an expression profile conforming to the various IL-5+ subpopulations noted above (14, 15). The continued development of peTh2 phenotypic markers that are amenable to immunohistochemcal detction, such as hPGDS, will facilitate understanding of their role in human disease. For the purposes of this review, we will use “peTh2” as a generic term referring to the various IL-5+ Th2 subpopulations described above. Conversely, the term “conventional” Th2 (cTh2) refers to a subpopulation of Th2 cells that is IL-5− or is negative for one of a variety of phenotypic markers associated with IL-5 expression, such as CD161, hPGDS, IL-17RB, or ST2.

## Murine Studies

Much of our understanding of peTh2 cells comes from the characterization of IL-5+ Th2 cells in mice. Immunological memory defines the adaptive immune system, and memory T cells can be subdivided into central memory (T_cm_), effector memory (T_em_), and resident memory (T_rm_) populations, reviewed in Ref. (16, 17). Of these, T_em_ lack CD62L (l-selectin) and can express a variety of chemokine receptors for homing to peripheral tissues. Nakayama and colleagues categorized subsets of memory Th2 cells according to their expression of CD62L and the Th1-associated chemokine receptor CXCR3. While all memory Th2 subsets expressed IL-4 and IL-13, only the CD62L^low^, CXCR3^low^ Th2 subpopulation was enriched for IL-5. Depletion of CD62L^low^, CXCR3^low^ Th2 cells attenuated eosinophilic inflammation and airway hyperresponsiveness in a mouse model of allergic airway inflammation. These findings indicate that CD62L^low^, CXCR3^low^ cells have peTh2 function. Later findings by this group demonstrated that memory Th2 cell pathogenicity depends on the IL-33/ST2 axis (18), raising interesting questions about the conditions required for the development of these cells (see discussion below under Section “Relationship of peTh2 to Other T Cell Subsets”).

Chemoattractant receptors mediate cell migration through lymphoid organs and peripheral tissues. A number of chemoattractant receptors have been associated with Th2 cells, including the prostaglandin D2 (PGD2) receptor CRTH2 (19), CCR3 (20), CCR4 (21), and CCR8 (22). Of these, Luster and colleagues (12) found that CCR8 defines an IL-5-enriched Th2 subset in both *in vitro-*differentiated and *ex vivo*-stimulated murine Th2 cells. CCR8 expression was associated with skin inflammation and tissue eosinophilia in a mouse model of chronic atopic dermatitis. In this model, CCL8-responsive CCR8+ Th2 cells showed increased proliferation and homing to allergen-sensitized skin. In line with these findings, the CCR8 ligand CCL8 was predominantly expressed in the skin and upregulated during allergic inflammation.

In summary, at least three studies in mice have characterized peTh2 cells as an IL-5-enriched subset of effector memory Th2 cells that have a distinct phenotype. These studies reveal a role for peTh2 cells in the pathogenesis of allergic inflammation. While mouse models have implicated peTh2 cells in allergic inflammation of the skin and airway, peTh2 cells have yet to be studied in murine models of allergic gut inflammation. Whether peTh2 cells are induced by or play a protective role in parasitic infection is unknown. Additional studies in models of allergic gut inflammation and parasite clearance will help further clarify the role of peTh2 cells in the type 2 immune response.

## Human Studies, Role in Human Disease

When human peTh2 cells were first identified, one obvious question was why had they not been previously described in the murine system? One reason for this unexpected delay in murine findings may be that most *in vivo* mouse experiments have a relatively short turn-around time that does not include sufficiently chronic antigen exposure to generate peTh2 cells in large numbers. In contrast, peTh2 may have been more easily identified in humans because of their role in diseases characterized by chronic antigenic exposure, including helminth infection (5, 23), eosinophilic gastrointestinal disease (EGID) (24), allergic asthma (25), and atopic dermatitis (10).

Early clues to the existence of peTh2 cells were seen in the restriction of IL-5 expression to a minority subpopulation of Th2 cells (5, 6). Further, whereas IL-4 and IFN-γ were modestly coexpressed, IL-5 and IFN-γ demonstrated no coexpression (8), suggesting that the expression of IL-5 was accompanied by the silencing of IFN-γ. It was not until a decade later, when polychromatic flow cytometry was employed, that clear populations of IL-5+ (IL-4+, IL-5+) and IL-5− (IL-4+, IL-5−) Th2 cells could be routinely identified (24). Subsequently, Upadhyaya et al. developed reagents and techniques to examine all three Th2 cytokines and demonstrate two major human Th2 subpopulations: a minority IL-5+ Th2 (IL-5+, IL-4+, IL-13+) and majority IL-5− Th2 (IL-5−, IL-4+, IL-13+) subset (9).

Notably, during *in vitro* differentiation of Th2 cells from naïve CD4 cells, IL-4 and IL-13 expression is rapidly acquired, whereas the acquisition of expression of all three Th2 cytokines requires multiple rounds of antigenic exposure (9, 13, 26). *Ex vivo* peTh2 cells are CD45RO+, CD45RA−, CCR7−, CD62L−, and CD27−, consistent with their being highly differentiated CD4 T cells that have undergone repeated antigenic exposure. Such repeated antigenic exposure is typical of many allergens. For example, peanut allergen-specific IL-5+ Th2 cells were found in EGID, whereas in peanut anaphylaxis, the peanut-specific Th2 response was almost entirely IL-5− Th2. In EGID, patients typically do not have immediate type hypersensitivity and have chronic exposure to dietary peanut antigen; in contrast, in peanut anaphylaxis exposure to peanuts is rare. Conversely, in patients with peanut anaphylaxis undergoing peanut antigen oral immunotherapy, EGID has been a well-described adverse outcome (27), suggesting that chronic antigen exposure drives the differentiation of IL-5− into IL-5+ Th2 cells.

## Regulation of Th2 Gene Expression in peTh2 Cells

The Th2 gene locus contains the genes for IL-4, IL-5, and IL-13 and is located on human chromosome 5q31 and mouse chromosome 11. The *IL4* and *IL13* genes are adjacent to each other, whereas *IL5* is 120 kb telomeric and in the opposite orientation. This gene arrangement, coupled with the finding that peTh2 cells are enriched for IL-5, suggests that epigenetic mechanisms may underlie peTh2 effector function. Histone modifications control chromatin structure and DNA accessibility to transcription factors; for example, H3K4 and H3K27 methylation marks are associated with gene activation and repression, respectively (28). In one study, peTh2 defined as CD62L^low^, CXCR3^low^ Th2 cells had increased H3K4me3 and decreased H3K27me3 binding to the *IL5* promoter, compared to other memory Th2 subsets (29). This same histone methylation pattern was seen in sorted human IL-5+ Th2 cells (9). These findings suggest that peTh2 cells are specifically licensed by an epigenetic program that results in the expression of *IL5*.

In addition to epigenetic regulation, current evidence suggests that peTh2 have a distinct transcriptional program. The Th2 master transcription factor GATA3 is required for both Th2 differentiation and for *IL5* and *IL13* expression (30). Interestingly, GATA3 increases with serial rounds of Th2 differentiation (9, 12) and is greatest in peTh2 cells (9, 29). Additionally, in peTh2 cells, GATA3 is preferentially associated with the *IL5* promoter, relative to cTh2 cells (9). Another regulator of peTh2 gene expression is the Th1-associated transcription factor eomesodermin. Eomesodermin is expressed at lower levels in CD62L^low^, CXCR3^low^ Th2 cells (peTh2) relative to other Th2 subpopulations (29). Through its interaction with GATA3, eomesodermin negatively regulates *IL5*, but not *IL4* or *IL13* expression by memory Th2 cells. In contrast to eomesodermin, T-bet expression is not differentially expressed in any specific Th2 subpopulation and knock-down of the T-bet gene (Tbx21) in Th2 cells did not affect Th2 cytokine expression. These data suggest a role for eomesodermin in inhibiting peTh2 development, in addition to its role in Th1 induction.

While several studies have shown that Th2 locus chromatin remodeling and Th2-associated transcription factors mediate peTh2 effector function, additional transcriptional mechanisms may also play a role. Wansley et al. recently found that the transcription factor retinoic acid receptor alpha (RARα) selectively regulates the proliferation and cytokine expression of IL-5+, but not IL-5−, human Th2 cells (31). This differential effect was attributed to a putative retinoic acid response element in the human *IL5* but not *IL4* or *IL13* promoters. Interestingly, vitamin A has been shown to promote the type 2 immune response *via* its metabolites binding RARα (32). These data suggest that vitamin A metabolites may amplify peTh2 effector function. In line with these findings, vitamin A supplementation correlated with disease severity in a murine model of asthma (33).

In summary, current evidence suggests that peTh2 cells have a unique epigenetic and transcriptional program underlying their effector function. The selective amplification of peTh2 cell activity by vitamin A metabolites raises the possibility that environmental factors can influence peTh2 cell responsiveness. Moving forward, the effect of diet on pathogenic type 2 inflammation may be a fruitful area of study.

## Relationship of peTh2 to Other T Cell Subsets

Several phenotypic and functional features distinguish peTh2 from cTh2 cells. Unlike cTh2 cells, peTh2 express hPGDS (10, 15). hPGDS is required for PGD2 production, and while mast cells are the dominant source of PGD2, we found that peTh2 cells produced PGD2 upon calcium ionophore stimulation (10). It is currently unknown whether and which physiological conditions induce PGD2 production by peTh2 cells. However, T cell receptor (TCR) stimulation failed to induce PGD2 in peTh2 (AMS, unpublished observations), raising the possibility that an innate stimulus drives hPGDS activity. Once produced, PGD2 binds to its receptor CRTH2, inducing Th2 cytokine production and chemotaxis of Th2 cells, type 2 innate lymphoid cells (ILCs), eosinophils, and basophils (34–36). Thus, peTh2 cells may propagate pathogenic type 2 inflammation *via* the hPGDS/PGD2/CRTH2 axis in both an autocrine and paracrine fashion.

Another difference between peTh2 and cTh2 cells lies in their effector function. Th2 cells have historically been identified by their expression of the Th2 cytokines IL-4, IL-13, and IL-5. When compared side-by-side, peTh2 cells not only express greater per-cell Th2 cytokines than their conventional counterparts but also have a distinct cytokine expression profile (9, 10). Indeed, several groups have found that IL-5 expression is restricted to peTh2 cells, whereas all Th2 subsets express IL-4 and IL-13 (9, 10, 13, 29). This differential cytokine expression is likely regulated by the epigenetic and transcriptional mechanisms outlined in the previous section and raises important questions about peTh2 development relative to cTh2 cells.

Several lines of evidence suggest that peTh2 are highly differentiated Th2 cells that arise from cTh2 cells after chronic antigen exposure. *In vitro*, Th2 differentiation can be induced by TCR stimulation of naïve T cells in Th2-polarizing conditions (26). While one round of differentiation induces cTh2 cells that express IL-4 and IL-13, *in vitro* generation of peTh2-like cells (that express IL-5, CCR8, and hPGDS in addition to IL-4 and IL-13) requires multiple rounds of differentiation (9, 10, 13). Notably, Paul and colleagues have demonstrated that the acquisition of ST2 expression and IL-33 responsiveness by Th2 cells (a peTh2 feature discussed below) similarly requires multiple rounds of *in vitro* differentiation (37). These findings are supported by *ex vivo* human studies in which peTh2 cells were uniformly CD27− (10), a pattern characteristic of highly differentiated memory effector T cells (38). Because Th differentiation is associated with chromatin remodeling at specific loci (39), the epigenetic signature of peTh2 cells (discussed in the previous section) further supports the notion that peTh2 are highly differentiated Th2 cells. Together, these studies suggest that peTh2 cell development and effector function require multiple rounds of differentiation that induce epigenetic modifications to Th2 cytokine loci. In support of this notion, Th2 cells that have undergone only two rounds of *in vitro* differentiation lack H3K4 methylation in the *IL5* promoter (40).

While peTh2-like cells can be generated *in vitro* through multiple rounds of Th2 differentiation, relatively little is known about the conditions required for their physiologic development *in vivo*. Recent studies, however, suggest that local inflammatory signals play a role. Thymic stromal lymphopoietin (TSLP) and IL-33 are epithelial-derived cytokines responsible for epithelial barrier maintenance (41, 42). In one study, TSLP-primed dendritic cells induced Th2 polarization and hPGDS expression (43). In another study, mice deficient in the IL-33R subunit ST2 failed to develop IL-5+ Th2 cells (44). Thus, local epithelial barrier disruption or pro-Th2 pathogen-associated molecular patterns that result in the release of TSLP and IL-33 may be an important pathway promoting peTh2 cell development.

Pathogenic effector Th2 can be further distinguished from cTh2 cells by their responsiveness to innate stimuli independent of canonical TCR activation. The innate and epithelial-derived cytokines IL-25, IL-33, and TSLP activate a type 2 immune response upon binding IL-17RB, the IL-33R complex, and the TSLPR complex, respectively, reviewed in Ref. (41, 42, 45). peTh2 cells not only express the receptors for but also produce Th2 cytokines upon stimulation by IL-25, IL-33, and TSLP (10, 12, 18, 37, 46). In one study, IL-33 induced H3K4 trimethylation and corresponding IL-5 production in memory Th2 cells *via* a p38/MAP kinase-dependent pathway (18). Whereas TCR stimulation induced IL-5 production by peTh2 only, IL-33 induced IL-5 production by all memory Th2 subsets. Together, these findings not only demonstrate that peTh2 cells have an innate-like program (see next section) but also implicate innate stimuli in the priming of peTh2 effector function.

The presence of peTh2 cells in the peripheral blood and at sites of allergic inflammation suggests that they have a pro-eosinophilic inflammatory chemotactic program (10, 12, 13). Indeed, peTh2 cells from subjects with EGID or atopic dermatitis expressed the Th2-associated chemokine receptor CCR3 and demonstrated enhanced chemotaxis to the CCR3 ligand eotaxin-1, whereas cTh2 cells did not (10). Furthermore, peTh2 cells from EGID and atopic dermatitis differentially expressed the gut and skin homing receptors α4β7 and CLA, respectively. In another study, peTh2 cells defined by their expression of CCR8 demonstrated increased homing to allergen-sensitized skin (12). In sum, peTh2 cells have an enhanced chemoattractant ligand and receptor program that facilitates their migration to sites of allergic inflammation.

The findings that peTh2 can be distinguished from cTh2 cells by their phenotype, enhanced effector function, innate responsiveness, and migratory capacity support a direct role for peTh2 cells in eosinophilic inflammation. This notion is further supported by the near perfect correlation of peTh2 cells with peripheral blood eosinophil counts in subjects with EGID and atopic dermatitis, suggesting that peTh2 cells drive eosinophilia in these diseases (10). peTh2 from these subjects have an activated phenotype and exhibit spontaneous proliferation relative to cTh2 cells. Together, these findings suggest that peTh2, and not cTh2 cells, mediate pathogenic type 2 inflammation. Whether peTh2 cells cause or result from chronic allergic inflammation, however, has yet to be formally investigated.

In summary, peTh2 are highly differentiated Th2 cells that likely develop from cTh2 cells through multiple rounds of Th2 polarization. Unlike cTh2 cells, peTh2 express IL-5 in addition to IL-4 and IL-13 and respond to innate stimuli including IL-25, IL-33, and TSLP. peTh2 have enhanced migratory function compared to cTh2 cells and localize to sites of allergic inflammation. Current evidence supports a model in which chronic antigen exposure at disrupted epithelial surfaces drive peTh2 cell differentiation, tissue trafficking, and consequent eosinophilic inflammation.

## Innate Function of peTh2, Similarities and Differences Between peTh2 and ILC2

Innate lymphoid cells are a recently characterized group of lymphocytes that lack the TCR but produce effector cytokines in patterns characteristic of Th cell subsets (47). While cTh2 cells require TCR stimulation for cytokine production, peTh2 can respond to stimuli independent of TCR activation, suggesting that they have innate-like qualities. Indeed, peTh2 share several functional features with ILC2, including responsiveness to IL-25, IL-33, and TSLP (10, 12, 18, 37, 47–49). Interestingly, stimulation by these innate and epithelial-derived cytokines induced comparable levels of IL-5 and IL-13 in both cell types (10). In addition to IL-5 and IL-13, peTh2 and ILC2 can also express IL-9 (10, 48).

Underlying the functional similarities between peTh2 and ILC2 is a shared transcriptional program. The Th2 master transcription factor GATA3 and RORα drive ILC2 development and effector function (50). In addition to expressing high levels of GATA3 (10, 29), peTh2 expressed greater levels of RORα compared to other memory Th2 populations (29). A shared transcriptional program may also explain the phenotypic similarities of peTh2 and ILC2, as both cell types express CRTH2, hPGDS, and the C-type lectin CD161 (10, 51).

The similarities between ILC2 and peTh2 in their effector function, transcriptional program, and phenotype raise important questions regarding their respective roles in the type 2 immune response. ILC2 are predominantly tissue-resident innate effectors cells (47) and are increased at sites of allergic inflammation (51, 52). While peTh2 cells have largely been characterized *ex vivo* from peripheral blood, they have a tissue homing phenotype and have been shown to localize to sites of allergic inflammation (10, 12, 13). Thus, both cell types are implicated in local allergic inflammation. Our current understanding of peTh2 supports a model in which chronic allergen exposure and type 2 inflammation induces the differentiation of peTh2 cells that have innate function.

Few studies have directly compared ILC2 vs. peTh2 cells in pathogenic type 2 inflammation (10), but some inferences can be made regarding their timing in the immune response. peTh2 cells require multiple rounds of direct antigen stimulation for their development, whereas ILC2 do not. Therefore, primary responses are likely to be dominated by ILC2 cells, whereas after chronic antigen exposure, differentiation and clonal expansion of peTh2 increases their number and innate functionality. Future studies will help further define the relative roles of peTh2 vs. ILC2 in the development and maintenance of allergic inflammation. Intriguingly, because of the numerous similarities between peTh2 and ILC2 cells, many therapeutic approaches will target both cell populations.

## Therapeutic Targeting of peTh2

The localization of pro-eosinophilic function to peTh2 cells suggests their unique features may represent a therapeutic target. Indeed, it is likely that the current generation of anti-cytokine monoclonal therapeutics is largely exerting their effect through activity on peTh2 cells or their products (e.g., Th2 cytokines). Both the anti-IL-5 monoclonals mepolizumab and reslizumab as well as the anti-CD124 monoclonal dupilumab demonstrate the greatest clinical activity in patients with the highest baseline eosinophils counts, patients who are also expected to have the greatest numbers of peTh2 cells (53–55).

Given the high levels of GATA3 expression by peTh2 and the GATA3 requirement for *IL5* expression, it is likely that the investigational anti-GATA3 DNAzyme SB010 will directly affect peTh2 cells. Similar to the findings seen with the anti-cytokine monoclonals, SB010 had its greatest activity in subjects with the highest baseline eosinophil counts (56).

As antagonists of IL-25, IL-33, and TSLP advance in clinical development, the specific role of these innate pro-Th2 cytokines will become clearer. The p38 mitogen-activated protein kinase is a downstream mediator of IL-33/ST2 signaling (18). Inhibition of p38 kinase activity specifically inhibits IL-33-induced IL-5 expression, suggesting it may be a druggable target for clinical development.

Pathogenic effector Th2 cells are notable for having both the biosynthetic machinery to synthesize PGD2 as well as the CRTH2 receptor to respond to PGD2. Although hPGDS inhibitors have been described in pre-clinical work, none has advanced thus far to clinical trials. In contrast, a number of CRTH2 inhibitors have been examined in clinical trials, the most promising being fevipiprant (57) and timapiprant (formerly OC000459) (58).

We have examined rapamycin as a potential anti-peTh2 drug. Notably, the rapamycin proliferation IC_50_ for peTh2 was shifted more than 2-fold vs. cTh2 and 100-fold vs. Th1 cells (59). Notably, the peTh2 IC50 was 0.1 nM, corresponding to serum concentrations <5% of that commonly used in transplant. peTh2 cells consistently demonstrated greater mechanistic target of rapamycin complex 1 (mTORC1) activity and greater susceptibility to mTORC1 inhibition than cTh2 or other CD4 T cell subsets. These data suggest that bioenergetic differences specific to the peTh2 subpopulation may allow their selective therapeutic targeting. Unfortunately, these promising *in vitro* findings were not translated in three EGID subjects who were treated with sirolimus for 8 weeks (CP, unpublished data).

## Conclusion

**Figure 1:**
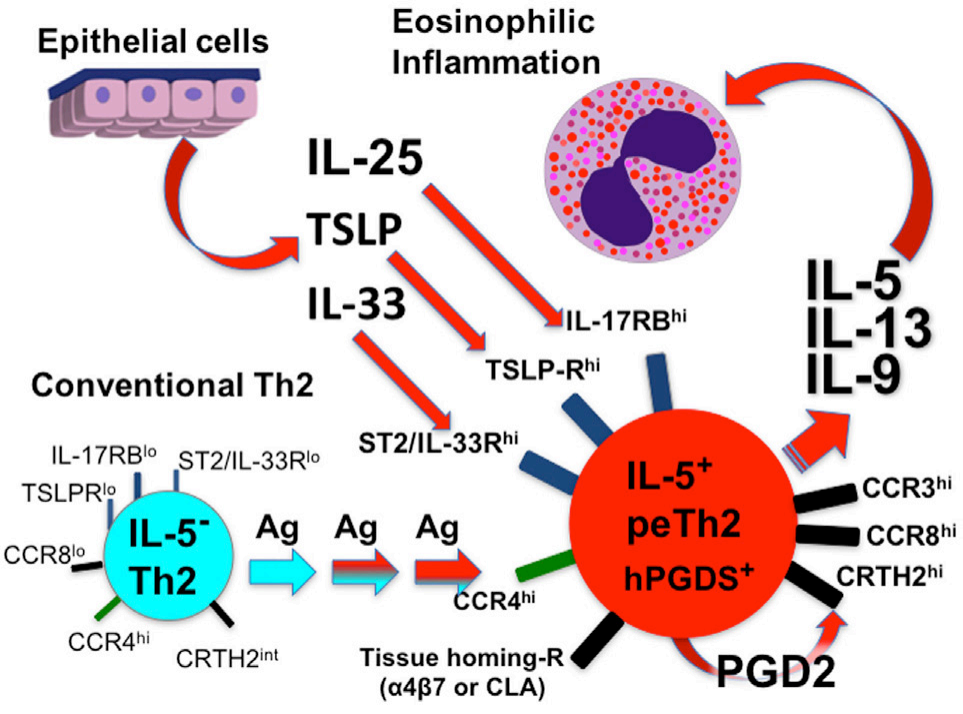
Development of peTh2 enhances Th2 function thereby driving chronic allergic inflammation.

Although many questions remain, the overwhelming evidence demonstrates two subpopulations of Th2 cells with distinct features (Table 1). Do peTh2 cells represent a separate Th2 subpopulation? If peTh2 cells are actually a distinct subpopulation, why does it matter? There is some evidence for a continuum of differentiation states between cTh2 and peTh2, which would argue against a clear dichotomy. However, under most conditions, the less differentiated cTh2 state is dominant, with peTh2 differentiation occurring only in specific pathological conditions (i.e., chronic antigen exposure). Thus, the acquisition of peTh2 function and pathology is a consequence of this chronic antigen exposure (Figure 1).

**Table 1:** Molecular features of pathogenic effector Th2 (peTh2) vs. conventional Th2 (cTh2).

Molecule	cTh2	peTh2	Reference
**Cytokines**			
IL-4	+++	++++	(9, 10, 13, 15, 29)
IL-5	+	++++	(9, 10, 13, 15, 29, 46)
IL-9	−	++	(10, 15)
IL-13	+++	++++	(9, 10, 13, 15, 29, 46)
IL-17	−	+	(10)
IFN-γ	+/−	−	(5, 9)
**Cytokine receptors**			
IL-17RB (IL-25R)	++	+++	(10, 15, 46)
IL-1RL1 (IL-33R, ST2)	++	+++	(10, 15, 18, 29, 46)
CRLF2 (TSLP-R)	++	++++	(10, 15)
**Chemoattractant/homing receptors**			
CCR3	+	+++	(10)
CCR4	+++	+++	(10)
CCR8	+	+++	(12, 13)
CXCR3	+	−	(10)
CRTH2	++	+++	(10)
**Transcription factors**			
GATA3	+++	++++	(9, 10, 12, 29)
T-bet	−	−	(11, 29)
Eomesodermin	+	−	(29)
**Other**			
Hematopoietic prostaglandin D synthase	+	++++	(10, 15)
CD27	++	−	(6, 9, 10, 15)
CD161	+	++++	(10, 15)

Given the importance of peTh2 cells in eosinophilic inflammatory diseases, investigational approaches that focus on peTh2 cells, rather than the entire Th2 compartment, are more likely to yield insights into these diseases. Similarly, therapeutic attempts to inhibit eosinophilic inflammation that focus on peTh2 cells will have greater potential for success.

## Author Contributions

AM-S and CP contributed equally to this work, including the conceptualization, writing, and editing.

## Author’s Note

This work was performed as an extracurricular activity outside of author CP’s cited affiliation.

## Conflict of Interest Statement

The author declares that the research was conducted in the absence of any commercial or financial relationships that could be construed as a potential conflict of interest.
